# Preserved endothelial function in human obesity in the absence of insulin resistance

**DOI:** 10.1186/1479-5876-11-263

**Published:** 2013-10-20

**Authors:** Mariam El Assar, Juan Carlos Ruiz de Adana, Javier Angulo, María Luz Pindado Martínez, Alberto Hernández Matías, Leocadio Rodríguez-Mañas

**Affiliations:** 1 Fundación para la Investigación Biomédica, Hospital Universitario de Getafe, Madrid, Spain; 2 Servicio de Cirugía General y del Aparato Digestivo, Hospital Universitario de Getafe, Madrid, Spain; 3 Instituto Ramón y Cajal de Investigación Sanitaria, Madrid, Spain; 4 Servicio de Anestesiología y Reanimación, Hospital Universitario de Getafe, Madrid, Spain; 5 Servicio de Geriatría, Hospital Universitario de Getafe, Madrid, Spain

**Keywords:** Insulin resistance, Obesity, Endothelial dysfunction, Nitric oxide, Oxidative stress, Inflammation, Mitochondria

## Abstract

**Background:**

Insulin resistance (IR) is frequently associated with endothelial dysfunction and has been proposed to play a major role in cardiovascular disease (CVD). On the other hand, obesity has long been related to IR and increased CVD. However it is not known if IR is a necessary condition for endothelial dysfunction in human obesity, allowing for preserved endothelial function in obese people when absent. Therefore, the purpose of the study was to assess the relationship between IR and endothelial dysfunction in human obesity and the mechanisms involved.

**Methods:**

Twenty non-insulin resistant morbid obese (NIR-MO), 32 insulin resistant morbid obese (IR-MO), and 12 healthy subjects were included. Serum concentrations of glucose, insulin, interleukin-6 (IL-6), tumor necrosis factor-alpha (TNF-α), resistin and adiponectin were determined. IR was evaluated by HOMA-index. Endothelium-dependent relaxation to bradykinin (BK) in mesenteric microvessels was assessed in wire myograph.

**Results:**

Serum IL-6, and TNF-α levels were elevated only in IR-MO patients while resistin was elevated and adiponectin reduced in all MO individuals. Mesenteric arteries from IR-MO, but not from NIR-MO subjects displayed blunted relaxation to BK. Vasodilatation was improved in IR-MO arteries by the superoxide scavenger, superoxide dismutase (SOD) or the mitochondrial-targeted SOD mimetic, mito-TEMPO. NADPH oxidase inhibitors (apocynin and VAS2870) and the nitric oxide synthase (NOS) cofactor, tetrahydrobiopterin failed to modify BK-induced vasodilatations. Superoxide generation was higher in vessels from IR-MO subjects and reduced by mito-TEMPO. Blockade of TNF-α with infliximab, but not inhibition of inducible NOS or cyclooxygenase, improved endothelial relaxation and decreased superoxide formation.

**Conclusions:**

Endothelial dysfunction is observed in human morbid obesity only when insulin resistance is present. Mechanisms involved include augmented mitochondrial superoxide generation, and increased systemic inflammation mediated by TNF-α. These findings may explain the different vascular risk of healthy vs unhealthy obesity.

## Background

Obesity represents one of the main health problems in modern societies, reaching epidemic proportions in developed countries [1]. Jointly to other consequences on health, obesity is one of the leading risk factors for cardiovascular disease (CVD). Endothelial dysfunction precedes in several years the clinical manifestations of CVD being a key event in its development [2]. A growing body of evidence reveals the presence of an altered endothelial vascular function manifested by a reduction in the endothelium-dependent vasodilatations in both animal models of obesity [3] and human obese subjects [4,5]. Frequently observed in obese patients, insulin resistance appears among the different pathogenic mechanisms leading to endothelial dysfunction. In fact, an impairment of endothelial function associated to insulin resistance has been documented in different vascular districts [3,4,6,7].

Although the high prevalence of both insulin resistance and endothelial dysfunction in obese people strongly suggests that these two usual features in the obese phenotype are causally linked, there is no available data in the literature directly addressing the implication of insulin resistance in the pathogenesis of endothelial dysfunction in human obesity. In fact studies showing impaired endothelial function in obese people have been carried out mainly by comparing endothelial function in vessels from obese subjects (without establishing differences between those who are sensitive or resistant to insulin action) to vessels from healthy adult subjects with normal weight (with preserved endothelial function). This is a key issue since clinical evidence suggests that there is a significant proportion (~30%) of obese individuals that are free from insulin resistance and does not manifest the panoply of cardiovascular diseases (CVD) usually observed in obese people [8]. This raises the hypothesis of a preservation of endothelial function and, consequently, vascular disease, when insulin resistance is lacking. Taken together, all these observations point to insulin-resistance as a major factor to explain the difference in CVD between both types of obese people.

Inflammation and reduced bioavailability of nitric oxide (NO) due in part to increased oxidative stress seem to play an outstanding role in the endothelial dysfunction associated to obesity [9,10]. Low-grade chronic vascular inflammation has been suggested to be a key event in the pathogenesis of endothelial dysfunction and CVD. Increased plasma concentrations of interleukin-6 (IL-6) and other inflammatory cytokines have been associated with the presence of endothelial dysfunction in obese patients [11]. In addition, tumor necrosis factor-alpha (TNF-α) and IL-6 levels have been shown to be independent predictors of coronary endothelial function [12]. Furthermore, inflammation and oxidative stress are intimately related processes. In fact, inflammatory cytokines reduce NO-mediated responses by increasing reactive oxygen species (ROS) production that results in decreased NO availability [13]. However, once again, no data is available in human vessels concerning the role of inflammatory cytokines or the contribution of oxidative stress to endothelial dysfunction in obese subjects with respect to the presence or absence of insulin resistance.

Although strong evidences support the relationship between insulin resistance and obesity as well as between insulin resistance and endothelial dysfunction, studies analyzing the specific role of insulin resistance in endothelial dysfunction associated to obesity are definitely lacking. Therefore, the aim of the present study was to determine the role of insulin resistance on endothelial function in obesity as a marker of early vascular damage that could provide a potential explanation for the different vascular outcomes in these two largely recognized subtypes of human morbid obesity: healthy and unhealthy. With this purpose we evaluated endothelial-dependent vasodilation in mesenteric microvessels from morbid obese individuals, with or without insulin-resistance, and the influence of inflammation and vascular ROS generation on these responses.

## Methods

### Study population

The present study included 52 morbid obese (MO) subjects with a body mass index (BMI) ≥ 40 kg/m^2^ aged between 25 and 65 years, who underwent bariatric surgery in the Hospital Universitario de Getafe with mixed techniques combining Roux-en-Y gastric bypass, vertical sleeve gastrectomy and adjustable gastric banding. Subjects with history or clinical evidence of CVD (congestive heart failure; cardiac and/or cerebrovascular ischemic disease) were excluded, but not those with cardiovascular risk factors like hypertension, type 2 diabetes, or dyslipidemia. Subjects were characterized as metabolic syndrome (MetS) if they met three of the following National Cholesterol Education Program Adult Treatment Panel III criteria as modified by the American Diabetes Association.

Insulin resistance was estimated by calculating the validated score for Homeostasis Model Assessment of Insulin Resistance (HOMA-IR) [14]. MO subjects were distributed into two groups depending on the HOMA-IR: non-insulin resistant (NIR; HOMA IR< 3.8; n=20) and insulin resistant (IR; HOMA-IR≥ 3.8; n=32). Cut-off point was previously defined for a Spanish population corresponding to the 90th percentile HOMA-IR value in non-diabetic adult subjects [15].

Twelve healthy non-obese subjects were recruited, as control group, among patients undergoing laparoscopic surgery procedures in our hospital (hiatus hernia repair, achalasia or cholecystectomies). Exclusion criteria for this control group were: BMI≥ 30 kg/m^2^; current or former smokers; hypertension (≥140/90 mmHg); diabetes; total cholesterol >200 mg/dl; history or clinical evidence of cardiovascular disease and any other condition that might interfere with the progress of the study. Written informed consent was obtained from the subjects who participated in the study. The study was approved by both the Ethics Committee and Research Committee of the Hospital Universitario de Getafe.

### Biochemical measurements

Blood samples were collected for measurement of serum fasting glucose, glycosylated hemoglobin (HbA_1C_), serum fasting insulin, serum lipid profile and C-reactive protein (CRP) in the Laboratory of Clinical Analysis of our hospital. HOMA-IR was calculated as described by Mathews et al. [16].

Circulating levels of TNFα, IL-6, adiponectin (R&D systems) and resistin (Bender MedSystems) were determined in serum by ELISA, following the manufacturer instructions, in the Research Unit Laboratory. All samples were assessed in duplicate.

### Measurement of vascular reactivity in isolated vessels

Small arteries (200–500 μm, approximately 2 mm length) were isolated from visceral fat obtained from MO or control subjects during laparoscopic surgery. The arteries were cleaned, and mounted as ring preparations on small vessel wire myographs, as described elsewhere [17,18]. Resting tension for the experiments was calculated to obtain an internal circumference equivalent to 90% of the tension of the vessels when relaxed *in situ* under a transmural pressure of 100 mmHg, using the Myo-Norm-4 program (Cibertec, Madrid, Spain). The vessels were contracted with K^+^125 mmol/l and the segments failing to produce a tension equivalent to a pressure of 100 mmHg were rejected. After a washout period, the arteries were contracted again with 25 mmol/l K^+^, which produced approximately 80% of the maximum response. When the contraction reached a plateau, the endothelium-dependent relaxation was assessed by adding increasing concentrations of bradykinin (BK; 10 nmol/l to 3 μmol/l). Vasodilatory effects of insulin (0.01 nmol/l to 3 μmol/l) were also evaluated. Responses to sodium nitroprusside (SNP; 1 nmol/l to 300 μmol/l) were used to test non endothelium-dependent relaxant responses.

In some experiments, the microvascular mesenteric segments were pretreated for 30 minutes before BK administration with the superoxide scavenger, bovine copper-zinc superoxide dismutase (SOD; 100 U/ml); the mitochondrial-targeted antioxidant mito-TEMPO (5 μmol/l); the NAD(P)H oxidase inhibitors, apocynin (100 μmol/l) or VAS2870 (10 μmol/l); the NO synthase (NOS) cofactor, tetrahydrobiopterin (BH_4_; 10 μmol/l); the non-selective cyclooxygenase inhibitor, indomethacin (10 μmol/l); the selective inducible NOS (iNOS) inhibitor, 1400 W (10 μmol/l); or the anti-TNFα monoclonal antibody, infliximab (100 μmol/l, Schering-Plough [Merck], Whitehouse Station, New Jersey). These experiments were systematically performed in a paired way, with parallel analysis of control and treated segments from the same subjects.

The experiments were performed in Krebs Henseleit solution (KHS), which was composed of (mmol/l): NaCl 115, CaCl_2_ 2.5, KCl 4.6, KH_2_PO_4_ 1.2, MgSO_4_.7H_2_O 1.2, NaHCO_3_ 25, glucose 11.1, and Na_2_EDTA 0.03. The solution was bubbled with a mixture of 95% O_2_ and 5% CO_2_ to maintain a pH of 7.4. Unless otherwise stated, all drugs used were purchased from Sigma (St Louis, MO, USA). Human recombinant insulin (Humulin R) was obtained from Eli Lilly, (Spain).

### Detection of vascular superoxide anion generation in microvessel segments

Formalin-fixed, paraffin-embedded transverse sections (5 μm thick) were mounted on polylysine-coated glass slides. The *in situ* production of superoxide anion was measured by means of the fluorescent dye dihydroethidium (DHE) as described previously [18]. Briefly, mesenteric arterial sections were incubated for 90 min at 37 °C with the fluorescent probe DHE (4 μmol/l; Calbiochem, Darmstadt, Germany). In the presence of superoxide anion, DHE is oxidized to ethidium that yields bright red fluorescence. After washing with PBS plus 0.1% Triton X-100, sections were mounted and visualized by fluorescence microscopy (Olympus BX51, Japan). The percentage of nuclei showing positive red signal with respect to total nuclei in arterial wall was determined with imaging software (McBiophotonics Image J, NIH, Bethesda, Maryland).

### Statistical analysis

Categorical variables were analyzed by the Chi-square test. Results from numerical variables were expressed as mean±SEM. The number of subjects (N) and the number of vascular segments (n) used for relaxation curves are indicated in each graph. Complete concentration-response curves to BK or to SNP in vessels from the participants were compared by a two-factors analysis of variance (ANOVA). Comparison of pD_2_, (which is defined as the negative logarithm of the concentration of BK required to obtain 50% of maximal relaxation) for each subject was statistically analyzed by a one-factor ANOVA followed by a Student-Newmann-Keuls test. This same analysis was also used to compare serum cytokine determinations and DHE staining. Student *t* test was used in all other comparisons. Pearson′s correlation coefficient was used to assess correlation among variables. Models of lineal regression were constructed, with the pD_2_ considered as the dependent variable. In all cases, a probability value of less than 5% was considered significant. Data were analyzed using version 20.0 of SPSS software (SPSS Corp, Chigaco, IL).

## Results

Insulin resistant morbid obese (IR-MO) subjects when compared to non insulin-resistant morbid obese (NIR-MO) group showed significantly larger waist circumference and higher levels of fasting insulin, HOMA-IR, HbA_1C_ and CRP, while HDL cholesterol levels were lower (Table 1). In addition, the percentage of MetS was significantly higher in IR-MO versus NIR-MO subjects. Comorbid conditions and treatments received by the patients at the time of surgery are shown in Table 1.

**Table 1:** Basal variables of morbid obese subjects

**Characteristic**	**Non**-**insulin resistant (N=20)**	**Insulin resistant (N=32)**	**P values**
**Clinical**			
Age (years)	42.9±2.9	42.3±1.7	0.837
Sex (M/F)	4/16	13/19	0.123
Body mass index (kg/m^2^)	46.2±1.0	47.4±1.1	0.427
Waist circumference (cm)	117.78±2.7	127.87±2.5	**0.012**
MetS (%)	33.3	64.3	**0.04**
Diabetes mellitus type 2 (%)	0.0	37.5	**0.002**
Hypertension (%)	20.0	37.5	0.183
Dyslipidemia (%)	25.0	21.9	0.795
Active smokers (%)	20.0	31.3	0.374
**Chemical**			
Fasting glucose (mg/dl)	89.6±1.9	97.3±3.8	0.128
HbA1c (%)	5.5±0.06	5.9±0.1	**0**.**023**
Total cholesterol (mg/dl)	180.5±6.5	164.5±7.1	0.129
HDL cholesterol (mg/dl)	45.3±2.7	37.0±1.8	**0**.**009**
LDL cholesterol (mg/dl)	107.7±6.8	99.9±6.9	0.449
Ratio (TC/HDL)	4.13±0.2	4.7±0.2	**0.169**
Triglycerides (mg/dl)	109.0±7.7	134.2±9.9	0.077
Fasting insulin (μU/ml)	10.1±0.6	29.8±3.0	**<0.001**
HOMA-IR	2.3±0.1	7.3±0.8	**<0.001**
CRP (mg/l)	5.6±0.9	10.9±1.3	**0.002**
**Treatment**			
Hypoglycemic drugs (%)	11.8	35.7	0.078
Hypotensive drugs (%)	29.4	35.7	0.664
Lipid-lowering drugs (%)	0.0	6.7	0.162

*M*= Male; *F*= Female; *MetS*= Metabolic syndrome; *HDL*= High-density lipoprotein; *LDL*= Low-density lipoprotein; *TC*= Total cholesterol; *HOMA*-*IR*= Homeostasis Model Assessment of Insulin Resistance; *CRP*= C-Reactive Protein. Significant differences are highlighted in bold.

Regarding biomarkers of inflammation and adipose tissue activity, TNF-α and IL-6 levels were increased in IR-MO patients when compared to control subjects, while there were no differences between NIR-MO and control subjects (Table 2). Finally, the levels of adiponectin were reduced and those of resistin increased, in both IR-MO and NIR-MO patients (Table 2).

**Table 2:** **Serum concentrations of biomarkers of inflammation and adipose tissue function**.

	**Control subjects**	**Non**-**insulin resistant morbid**	**Insulin resistant morbid obese subjects (n=27)**
	**(n=12)**	**obese subjects(n=20)**	
**Adiponectin (μg/ml)**	9.47±0.12	6.27±0.49 ^¶^	5.09±0.63 ^†^
**Resistin(ng/ml)**	1.95±0.22	3.85±0.54 ^*^	4.33±0.56 ^*^
**TNF-α (pg/ml)**	4.61±0.45	5.65±0.39	6.41±0.47 ^*^
**IL-6 (pg/ml)**	1.24±0.19	2.81±0.37	4.81±0.78 ^¶, ‡^

*TNF*-α= Tumor necrosis factor alpha; *IL*-*6*= Interleukin 6. * p< 0.05; ^¶^ p< 0.01; ^†^ p< 0.001 vs control subjects. ^‡^ p< 0.05 vs non-insulin resistant morbid obese subjects.

### Endothelial dysfunction is associated with insulin resistance in morbid obese subjects

**Figure 1:**
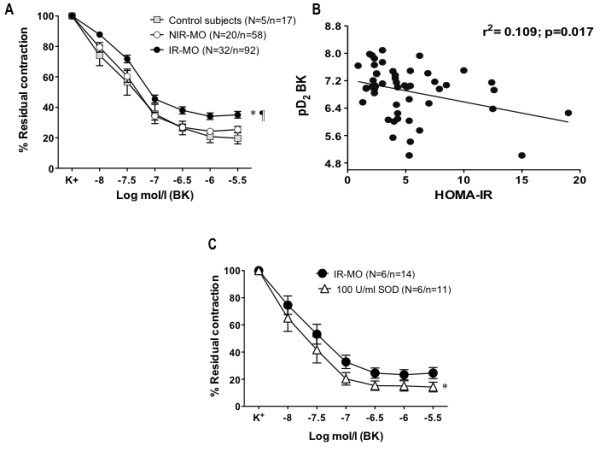
**Insulin resistance is related to endothelial dysfunction in morbid obesity. (A)** Vasodilation to bradykinin (BK) in mesenteric arterial segments derived from control subjects, non-insulin resistant (NIR-MO) and insulin resistant morbid obese (IR-MO) subjects. Data are expressed as mean±SEM of the percentage of the contraction elicited by K^+^. N= the number of subjects/ n= the number of vascular segments. ^*^p< 0.001 vs NIR-MO and ^¶^p< 0.001 vs control subjects by two-factors ANOVA. **(B)** Negative correlation between Homeostasis Model Assessment of Insulin Resistance (HOMA-IR) score and pD_2_ values for BK. Each point represents the averaged pD_2_ values of segments from one single subject. **(C)** Effect of preincubation with the superoxide dismutase (SOD; 100 U/ml) on the relaxant responses to BK in mesenteric arteries from IR-MO. ^*^p< 0.05 vs IR-MO subjects by two-factors ANOVA.

Small mesenteric arteries from NIR-MO did not show significant differences in the BK-evoked vasodilation when compared to control non-obese subjects (Figure 1A). By opposite, a significant reduction in the endothelium-mediated vasodilation induced by BK was observed in mesenteric microvessels obtained from IR-MO group when compared to those from control or NIR-MO subjects (Figure 1A). To avoid a potential bias due to the comparison of the vascular responses instead of comparing the response in the subjects, we confirmed these results assessing the differences between the pD_2_ values for BK averaged from all the vessels from each subject by group (control, NIR-MO and IR-MO). This approach yielded very similar results to that observed when we analysed the response of the vessels. pD_2_ for BK were 7.23±0.20 (N=12), 7.27±0.11 (N=20) and 6.69±0.14 (N=32) for control, NIR-MO and IR-MO, respectively (p < 0.01 IR-MO vs. NIR-MO or control subjects by one-factor ANOVA followed by Student-Newmann-Keuls).

Accordingly, the *in vitro* vasorelaxant response to BK in microarteries derived from MO subjects decreased as the HOMA-IR index increased with a clear correlation between the HOMA-IR index and the BK pD_2_ values (Figure 1B). Furthermore, among all factor studied (Age, BMI, waist circumference, fasting glucose, HbA_1C_, total cholesterol (TC), HDL and LDL-cholesterol, TC/HDL ratio, triglycerides, CRP and HOMA-IR), only HOMA-IR (p= 0.017) was associated to BK pD_2_ values.

The addition of insulin (0.01 nmol/l to 3 μmol/l) to the organ bath evoked a very weak relaxation in isolated mesenteric arteries derived from obese and control subjects and no differences were observed among those subjects (E_max_: 16.0%±10.5 vs 11.2%±3.0 and vs 12.5%±3.2 for control, NIR-MO and IR-MO subjects, respectively; p=n.s.).

No differences were found in the non-endothelium dependent relaxations evoked by SNP in vessels from IR-MO people as compared with NIR-MO subjects (pD_2_ 5.59±0.19 vs 4.96±0.25 and vs 5.10±0.30 for control, NIR-MO and IR-MO subjects, respectively; p=n.s.).

### Oxidative stress contributes to endothelial dysfunction in IR-MO subjects

The contribution of superoxide anions to endothelial dysfunction in IR-MO subjects was confirmed. Preincubation of the microarteries from these subjects with the superoxide scavenger, SOD (100 U/ml), improved the endothelial-dependent relaxation to BK (Figure 1C), while it did not modify the responses to BK in vessels from NIR-MO subjects.

**Figure 2:**
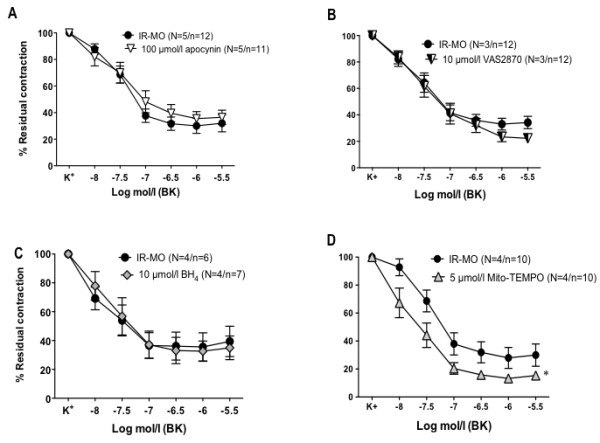
**Sources of superoxide anions in mesenteric arteries of morbid obese subjects with insulin resistance (IR-MO).** Effects of preincubation with the NADPH oxidase inhibitors, apocynin (100 μmol/l) **(A)**; or VAS2870 (10 μmol/l) **(B)**; the NOS cofactor, tetrahydrobiopterin (BH_4_; 10 μmol/l) **(C)**; and the SOD mimetic targeted to the mitochondria, mito-TEMPO (5 μmol/l) **(D)** on the relaxant response to bradykinin in isolated mesenteric microvessels from IR-MO subjects. Data are expressed as mean±SEM of the percentage of the contraction elicited by K^+^. N= the number of subjects/n= the number of vascular segments. ^*^p< 0.001 vs IR-MO by two-factors ANOVA.

With respect to the possible sources of superoxide production, the roles of NADPH oxidase, NOS uncoupling and mitochondria were analyzed. The NADPH oxidase inhibitor, apocynin (100 μmol/l) did not significantly modify BK-induced relaxations in mesenteric microvessels from IR-MO subjects (Figure 2A). Similar results were obtained when using the novel specific NADPH oxidase inhibitor, VAS-2870 (10 μmol/l) (Figure 2B). The NOS cofactor, tetrahydrobiopterin (BH_4_, 10 μmol/l) also failed to improve BK-induced vasodilations in arteries from IR-MO subjects (Figure 2C). In contrast, the SOD mimetic targeted to the mitochondria, mito-TEMPO (5 μmol/l), caused a significant improvement of endothelium-dependent vasodilation of arteries from IR-MO subjects (Figure 2D). None of these treatments modified the vasodilatations to BK in microvessels derived from NIR-MO (pD_2_: 7.03±0.55 vs. 7.07±0.43; 7.01±0.06 vs. 7.33±0.20; 7.20±0.31 vs. 6.89±0.42; 7.00±0.41 vs. 6.98±0.29) for VAS-2870, apocynin, BH_4_, and mito-TEMPO, respectively).

**Figure 3:**
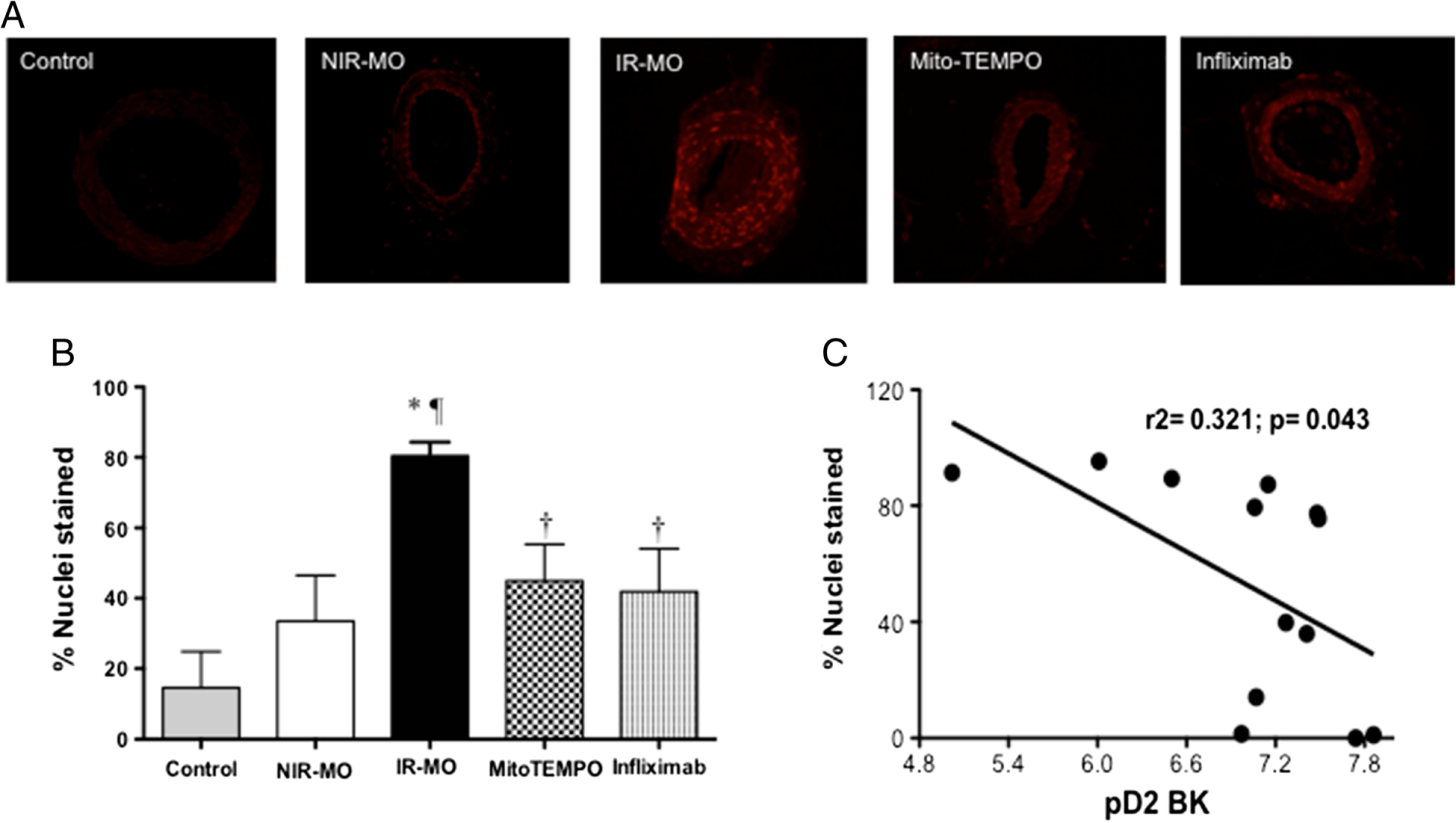
**Detection of superoxide generation in vessel segments from morbid obese subjects without insulin resistance** (**NIR**-**MO**) **and with insulin resistance (IR-MO) and the effect of mitochondria antioxidant, mito-TEMPO and the anti-TNF-α, infliximab on superoxide generation in IR-MO microarteries.** Representative photomicrographs **(A)** and quantitative analysis **(B)** of the staining (red signal) with the superoxide-detecting fluorescent probe, dihydroethidium (DHE), in mesenteric microvessels derived from control, NIR-MO and IR-MO subjects treated or not with the superoxide scavenger targeted to the mitochondria, mito-TEMPO (5 μmol/l), and the anti-TNF-α, infliximab (100 μmol/l) on superoxide generation in IR-MO vessels. Original magnification is (20X). ^*^p< 0.001 vs control subjects; p < 0.01 vs NIR-MO subjects; ^†^ p< 0.05 vs IR-MO subjects respectively. Each column represents the mean±SEM of 4 to 6 experiments. Panel **(C)** shows the correlation between the percentages of DHE positive nuclei and pD_2_ values for BK.

Consistently, superoxide production, assessed by DHE fluorescence, was clearly appreciated in vascular wall sections from IR-MO subjects, while the fluorescence signal was very weak or absent in vessels from NIR-MO and control non-obese subjects (Figure 3A). Quantification of staining revealed a significant increase in superoxide production in arteries from IR-MO with respect to the other two groups (Figure 3B). In fact, superoxide generation negatively correlated with pD_2_ for BK in mesenteric microvessels (Figure 3C). Furthermore, the improvement of endothelium-dependent vasodilation with mito-TEMPO was accompanied by a significant reduction of superoxide generation in arterial wall from IR-MO subjects (Figure 3A-B).

### Inflammatory cytokine, TNF-α, participates in the impairment of endothelial vasodilation in IR-MO subjects

**Figure 4:**
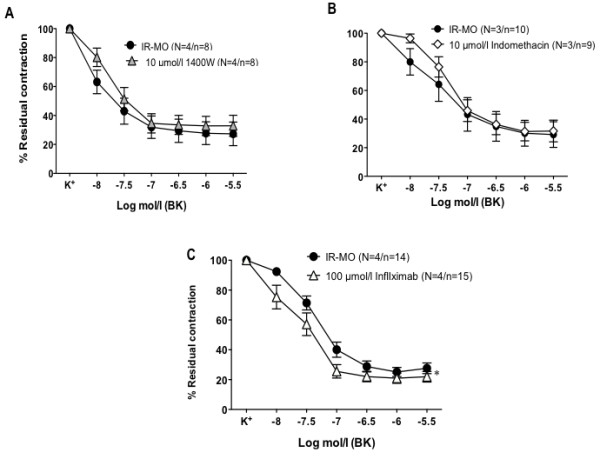
**Role of inflammation on endothelial dysfunction in morbid obese subjects with insulin resistance (IR-MO).** Effects of preincubation with the iNOS inhibitor, 1400W (10 μmol/l) **(A)**, the COX inhibitor, indomethacin (10 μmol/l) **(B)**, and the anti-TNF-α, infliximab (100 μmol/l) **(C)** on the relaxant response to bradykinin (BK) in isolated mesenteric microvessels from IR-MO. Data are expressed as mean±SEM of the percentage of the contraction elicited by K^+^. N= the number of subjects/n= the number of vascular segments. ^*^p< 0.05 vs IR-MO subjects by two-factors ANOVA.

In mesenteric arteries derived from IR-MO subjects, neither iNOS selective inhibitor 1400W (10 μmol/l) (Figure 4A), nor the cyclooxygenase inhibitor indomethacin (10 μmol/l) (Figure 4B) modified the impaired endothelial function observed in these subjects. By contrast, the anti-TNF-α, infliximab (100 μmol/l), improved the relaxation to BK in arteries from IR-MO subjects (Figure 4C). Interestingly, TNF-α blockade with infliximab also resulted in decreased generation of superoxide in these arteries (Figure 3A-B). None of those three compounds had any effect on endothelium-dependent vasodilation in microvessels derived from NIR-MO (pD_2_ for BK: 7.67±0.26 vs. 7.57±0.48, 7.23±0.29 vs. 7.00±0.55 and 7.47±0.26 vs. 7.59±0.01, for 1400W, indomethacin and infliximab, respectively).

## Discussion

The main finding of the present study is that endothelial dysfunction is present only in vessels from obese people with insulin resistance, suggesting that the association between morbid obesity and endothelial dysfunction is linked to the presence of insulin resistance. Such alteration is mediated by increased mitochondrial oxidative stress and by systemic pro-inflammatory cytokines (mainly TNF-α). The presence of other cardiovascular risk factors commonly found in obesity does not seem to explain the impact of IR on endothelial function in MO subjects, since no significant differences were observed between both groups of obese subjects. In addition, only insulin resistance (HOMA-IR) remained associated with endothelial function in obese subjects after adjustment.

Furthermore, endothelial dysfunction associated to insulin resistance in obese subjects does not seem to rely on a defective vasodilatory action of insulin since, in contrast to BK-induced responses, insulin-induced vasodilatory responses were not different between MO subjects with or without IR.

To our knowledge this is the first study describing this phenomenon in human beings, although recent results obtained in the microcirculation of obese mice support our hypothesis [19]. Other previous studies had suggested a relationship between endothelial function and insulin, HOMA-IR and triglycerides in obese subjects [20] but without establishing a clear causal relationship since the design of the study did not allow for comparing people with insulin-resistance versus people without insulin-resistance. Supporting our findings there is another study showing that IR and not BMI is the major determinant in the impairment of flow mediated dilation in morbid obese subjects [21]. In this sense, an improvement of the endothelial function after weight reduction in obese patients correlates with reduction in HOMA index rather than with reduction in body weight [22].

Although not reaching statistical significance, a larger percentage of IR-MO subjects were taking hypoglycemiants, being the vast majority metformin and pioglitazone. It is unlikely that this fact could have influence on the impaired endothelial dysfunction in IR-MO group since these agents have been reported to even improve endothelial vasodilation [23]. Even more, to discard a possible bias in the results due to the inclusion of twelve patients with the diagnosis of type 2 diabetes in the group of IR-MO patients, we made additional analysis of the data after excluding diabetic patients. The differences between the groups (NIR-MO and IR-MO) remained without changes. Furthermore, when diabetic type 2 patients were excluded, the HOMA-IR still significantly correlated with poorer BK-induced vasodilation, therefore reinforcing the hypothesis that views insulin resistance as a main determinant of endothelial dysfunction in obese subjects independently of the presence of diabetes. In addition, the percentage of metabolic syndrome is more frequent in IR-MO when compared to NIR-MO subjects, supporting the correct classification of the patients.

Several pathophysiological mechanisms are possibly involved in the interactions among obesity, insulin resistance and endothelial dysfunction [24]. In this sense, microarteries from IR-MO subjects generate higher amounts of intravascular ROS than those from NIR-MO or control subjects, as demonstrated by DHE staining. In fact, the superoxide scavenger, SOD, caused a significant improvement of endothelium-dependent relaxation only in mesenteric arteries from IR-MO subjects, suggesting that increased superoxide production contributes to endothelial dysfunction in IR-MO vessels by reducing NO availability. The involvement of superoxide excess in endothelial dysfunction in obesity is supported by demonstration of increased vascular superoxide generation in obese subjects [25] and in animal models of obesity [26] and by the potentiating effect of superoxide scavengers on ACh-induced relaxations in aortic and mesenteric arteries from obese animals [27,28] and in mesenteric microarteries from obese patients [25]. Once again, these studies did not establish differences based on the presence or absence of insulin resistance. Present results suggest that increased superoxide production and subsequent NO inactivation is associated to IR rather than to obesity itself, since elevated vascular superoxide generation and improving vasodilation in response to SOD are not detected in obese subjects without IR, who do not present altered endothelial vasodilation although they have a BMI similar to those with IR.

Several sources of ROS could contribute to the increased superoxide production in the vasculature of IR-MO subjects. In contrast to that observed in the vasculature of obese subjects in other studies [25], we did not find evidence for the possible implication of NADPH oxidase since its inhibitors, apocynin and VAS2870, did not improve endothelial vasodilation in mesenteric microarteries from IR-MO subjects. NOS uncoupling can also act as an endothelial source of superoxide. Reduced availability of its cofactor, BH_4_, in pro-oxidant conditions has been proposed as a mechanism responsible for eNOS uncoupling [29]. The failure of BH_4_ to modify vasodilatations suggests that this does not account for the reduced NO availability associated to IR in MO subjects. The improvement of endothelial vasodilation caused by the mitochondria-targeted superoxide scavenger, mito-TEMPO, in vessels from IR-MO subjects suggests that the origin of the impaired NO-mediated responses in IR associated with obesity is an increased mitochondrial superoxide production that limits NO availability. Mito-TEMPO has been demonstrated to specifically scavenge mitochondrial superoxide in endothelial cells and vascular tissue [30,31]. Mitochondria is considered the major intracellular site of superoxide production generated from electron leaks in the mitochondrial electron transport system [32]. When metabolic alterations lead to inefficient nutrient oxidation, mitochondria increases the production of superoxide and other ROS [32]. There is substantial evidence proposing the importance of these mitochondrial ROS in the pathophysiology of the vascular damage linked to diabetes and insulin resistance [33]. In fact, clinical evidence has associated insulin resistance with mitochondrial dysfunction [34,35]. Although it is difficult to ascertain whether defects in mitochondria occur before or after the onset of insulin resistance [33], it has been demonstrated that induction of mitochondrial superoxide production results in insulin resistant adipocytes [36].

On the other hand, the elevated levels of CRP and the pro-inflammatory cytokines, TNF-α and IL-6, reflect the presence of low-grade inflammation in obese subjects with IR that could contribute to endothelial dysfunction. In this sense, IL-6 and TNF-α have been demonstrated to impair endothelial dilation [37,38]. Furthermore, the effects produced by infliximab on endothelial vasodilatation in obese subjects, only when IR is present, suggest a causal role of inflammatory cytokines, TNF-α at least, in the endothelial impairment exhibited by the vessels from these subjects. Previous evidences showing that blockade of TNF-α with infliximab results in the recovery of impaired endothelial vasodilation in omental vessels from obese subjects [25] support this hypothesis. CRP levels did not show any correlation with pD2, although we cannot definitively exclude its participation.

The impact of systemic inflammatory cytokines in endothelial dysfunction does not seem to be mediated by up-regulation of conventional local inflammatory pathways, since both inhibitors of iNOS and COX did not improve endothelial relaxation. Deleterious effects of pro-inflammatory cytokines on endothelial function in obese patients could be also mediated by oxidative stress generation since these effects can be reversed by superoxide scavengers [25,39]. Furthermore, exposure to TNF-α stimulates mitochondrial superoxide production in mouse adipocytes [36] and human retinal endothelial cells [40]. This is consistent with the reduction of superoxide production observed in arteries from IR-MO subjects after the treatment with infliximab. Thus, systemic inflammation and mitochondrial superoxide production could be two closely related or even interdependent mechanisms mediating endothelial dysfunction in insulin resistance associated with obesity.

Obese subjects exhibit increased circulating levels of pro-inflammatory resistin and decreased levels of the anti-inflammatory cytokine, adiponectin, irrespective of the state of IR, which is in agreement with previous studies done in obese patients [41]. Then, increased adiposity is associated with adipose tissue inflammation and dysregulation of adipokines but this condition is related to obesity per se independently of the insulin resistance state, and, therefore, it does not seem to be determinant for the development of endothelial dysfunction.

We recognize that one limitation of the study is that vascular function has been evaluated in subjects with severe obesity so caution is required when any extrapolation from the present is made to overweight or non-severe obese subjects. Another potential limitation is that the present findings have been achieved based on an *in vitro* approach where endothelial function has been assessed in isolated microarteries segments. Therefore further *in vivo* studies (carotid artery ultrasound and/or endo-peripheral arterial tone) are needed to confirm our findings. However, a recent study showing *in vivo* a strong dependence of endothelial dysfunction on insulin resistance in obese individuals supports our findings [21].

## Conclusions

The present results reveal the determinant role of insulin resistance in the impairment of endothelium-dependent vasodilation in morbid obese patients. Endothelial dysfunction is produced by means of a reduced NO availability as a result of increased vascular superoxide generation by mitochondria. Moreover, the inflammatory mediator TNF-α contributes to the impaired vasodilation associated to IR in obese subjects. These findings may help to explain the link between obesity, IR and vascular dysfunction and provide targets for specific intervention aimed at delaying or preventing the appearance of vascular disease.

## Abbreviations

BH4: Tetrahydrobiopterin; BK: Bradykinin; BMI: Body mass index; CRP: C-reactive protein; CVD: Cardiovascular disease; DHE: Dihydroethidium; HDL: High density lipoprotein; HOMA-IR: Model Assessment of Insulin Resistance; IL-6: Interlukin-6; IR: Insulin resistant; MetS: Metabolic syndrome; MO: Morbid obese; NIR: Non-insulin resistant; NO: Nitric oxide; ROS: Reactive oxygen species; SNP: Sodium nitroprusside; SOD: Superoxide dismutase; TC: Total cholesterol; TNF-α: Tumor necrosis factor-alpha.

## Competing interests

The authors declare that they have no competing interest.

## Authors' contributions

MEA designed and performed experiments, analyzed and interpreted data, and wrote the manuscript. JCRDA, MLPM and AHM provided specimen, and drafted the manuscript. JA performed experiments, analyzed and interpreted data, and drafted the manuscript. LRM conceived the study, designed, interpreted data and drafted the manuscript. MEA and LRM critically discussed the paper. All the authors gave their final approval for the submission of the manuscript.
